# Improving the transparency and integrity of scientific reports on health. New instructions for authors!

**DOI:** 10.1590/1516-3180.2019.1372100419ap

**Published:** 2019-05-08

**Authors:** Álvaro Nagib Atallah, Patrícia Logullo

**Affiliations:** I MD, PhD. Full Professor and Head of the Discipline of Emergency Medicine and Evidence-Based Medicine, Universidade Federal de São Paulo (UNIFESP), and Director of Cochrane Brazil, São Paulo (SP), Brazil.; II BSc, MSc, PhD. Researcher at the EQUATOR Network, University of Oxford, Oxford, United Kingdom.

Healthcare practice should be based on the best available evidence, coming from rigorous research methodologies.^1^ Improvement of practice, going from doing no harm to incorporation of new technologies in public assistance, therefore requires access to robust scientific evidence, through the availability of good and complete research reports. However, what we see today is that access to high-quality evidence is somewhat hampered by poor reporting.

More than 80% of clinical trials and observational studies published today fail to report one or more important feature of their methodology or results.^2^ Inconsistent, biased, incomplete and inaccurate reports are published in the medical literature every day.^3^^,^^4^ However, we have resources to fight this battle: just as we have clinical guidelines for practice, we also have reporting guidelines to help authors to write and publish better research reports. These are the articles that systematic reviewers use to synthetize the evidence and inform practice.

Reporting guidelines have existed for more than 20 years now. However, adherence to them by authors, peer reviewers and journals has been modest and slow.^2^^,^^5^^,^^6^^,^^7^ The São Paulo Medical Journal has taken a step forward in the direction of improving transparency, quality and integrity of scientific reporting within the field of health research, in this issue. We are now publishing new Instructions for Authors in which we emphasize the need to adhere to reporting guidelines, and we will require all authors to submit complete reports.

In this new version of the São Paulo Medical Journal Instructions for Authors, we have taken into consideration the main reporting guidelines and principles of good reporting that are available. There is at least one reporting guideline for each main study design type, and they are all available through the EQUATOR Network website (http://www.equator-network.org).

The EQUATOR (Enhancing the Quality and Transparency of Health Research) Network is an international initiative that has been promoting the use of reporting guidelines since 2008. The UK EQUATOR Centre curates and maintains a very large and searchable database of reporting guidelines, provides toolkits for writing and organizes many training initiatives. It thus makes available a large amount of material to support author within the mission of better reporting.^5^^,^^8^ These instructions are very useful for everyone really interested in developing skills in clinical research.

Good reporting encompasses research reports that are clear and transparent, and that empower reproducibility. Clarity means being unambiguous and not allowing more than one interpretation. This is an essential feature within health research reporting, in which any misinterpretation can potentially prove fatal. Transparency means reporting everything, even bad news or methods that failed, which is broadly supported through efforts to encourage registration of clinical trials and systematic reviews prior to study commencement. Reproducibility requires details: again, reporting everything that was done and found, so that other researchers can repeat experiments. These are all essential features of scientific reporting.^9^

In our new Instructions for Authors, we have also considered the latest revision of the Recommendations by the International Committee of Medical Journal Editors (ICMJE), released in December 2018.^10^ In this latest revision, sensitive issues like authorship, plagiarism (andself-plagiarism), conflicts of interest and other matters are now addressed more explicitly by the Committee.^11^ Many of these issues are problems that our staff have been dealing with for a long time.Our new Instructions for Authors clearly set out what authors need to know about our submission requirements, what they should expect from the Journal and also what the Journal expects from them. As the late Douglas Altman said, “Readers should not have to infer what was probably done, they should be told explicitly”.^12^
